# THE VAXED PROJECT: An Assessment of Immunization Education in Canadian Health Professional Programs

**DOI:** 10.1186/1472-6920-10-86

**Published:** 2010-11-26

**Authors:** Lorine P Pelly, Donna M Pierrynowski MacDougall, Beth A Halperin, Robert A Strang, Susan K Bowles, Darlene M Baxendale, Shelly A McNeil

**Affiliations:** 1 Canadian Center for Vaccinology, IWK Health Centre, Dalhousie University, Dr. Richard B. Goldbloom RCC Pavilion, 4th Floor, 5850/5980 University Avenue, PO BOX 9700, Halifax, NS, B3K 6R8, Canada; 2 St. Francis Xavier University, Canadian Center for Vaccinology, IWK Health Centre, Dalhousie University, Dr. Richard B. Goldbloom RCC Pavilion, 4th Floor, 5850/5980 University Avenue, PO BOX 9700, Halifax, NS, B3K 6R8, Canada; 3 Nova Scotia Department of Health Promotion and Protection; Maritime Center, 1505 Barrington St., PO BOXC 2734, B3J 3P7, Canada; 4 Department of Medicine, QEII Health Sciences Centre, School of Pharmacy, Dalhousie University; Halifax, Nova Scotia, B3 H 3J5, Canada; 5 Department of Medicine, QEII Health Sciences Centre, Canadian Center for Vaccinology, IWK Health Centre, Dalhousie University, Dr. Richard B. Goldbloom RCC Pavilion, 4th Floor, 5850/5980 University Avenue, PO BOX 9700, Halifax, NS, B3K 6R8, Canada

## Abstract

**Background:**

Knowledge & attitudes of healthcare providers (HCP) have significant impact on frequency with which vaccines are offered & accepted but many HCP are ill equipped to make informed recommendations about vaccine merits & risks. We performed an assessment of the educational needs of trainees regarding immunization and used the information thus ascertained to develop multi-faceted, evaluable, educational tools which can be integrated into formal education curricula.

**Methods:**

(i) A questionnaire was sent to all Canadian nursing, medical & pharmacy schools to assess immunization-related curriculum content (ii) A 77-item web-based, validated questionnaire was emailed to final-year students in medicine, nursing, & pharmacy at two universities in Nova Scotia, Canada to assess knowledge, attitudes, & behaviors reflecting current immunization curriculum.

**Results:**

The curriculum review yielded responses from 18%, 48%, & 56% of medical, nursing, & pharmacy schools, respectively. Time spent on immunization content varied substantially between & within disciplines from <1 to >50 hrs. Most schools reported some content regarding vaccine preventable diseases, immunization practice & clinical skills but there was considerable variability and fewer schools had learning objectives or formal evaluation in these areas. 74% of respondents didn't feel comfortable discussing vaccine side effects with parents/patients & only 21% felt they received adequate teaching regarding immunization during training.

**Conclusions:**

Important gaps were identified in the knowledge of graduating nursing, medical, & pharmacy trainees regarding vaccine indications/contraindications, adverse events & safety. The national curriculum review revealed wide variability in immunization curriculum content & evaluation. There is clearly a need for educators to assess current curricula and adapt existing educational resources such as the Immunization Competencies for Health Professionals in Canada.

## Background

Vaccines are undeniably one of the most important health advances of the past century. Despite proven impact on human health and longevity, many vaccines are under-utilized. The literature has clearly documented low levels of compliance with established immunization guidelines in a variety of settings [1-3]. Reasons for sub-optimal compliance and "missed-opportunities" are multi-factorial. Public and provider confidence in vaccine efficacy, concern about potential side-effects, and lack of knowledge about vaccine contraindications are common reasons for non-compliance and have been shown to contribute to lower immunization coverage in both adult and pediatric populations [4-8]. Low rates of influenza vaccination have also been reported in health care workers. In 2007-2008 only 54% of direct care providers and 37% of support staff in acute care facilities in Nova Scotia received influenza vaccination; this rate is similar to published rates for providers in the United States and Canada [9-11].

The public views health care providers as credible and trusted sources of vaccine recommendations. Many individuals cite the recommendation of their physician or nurse as the most important factor governing their decision to either become vaccinated themselves, or have their child vaccinated, and positive attitudes of health professionals have been shown to correlate with higher vaccination coverage rates [7,12]. Despite the importance of provider endorsement and advice regarding vaccines, many healthcare providers report discomfort discussing misconceptions about adverse events following immunization with their patients and admit to being unsure about the relationship between vaccines and certain chronic diseases [13,14].

To our knowledge no studies have examined the provision of immunization-related education during health care professional training in Canada nor the knowledge, attitudes, beliefs and behaviors of trainees graduating from Canadian health professional programs. In this study we undertook a comprehensive assessment of the needs of healthcare professional trainees regarding formal immunization education. This needs assessment will form the platform upon which to develop multi-faceted, evaluable, interprofessional educational interventions, which can be integrated into formal education curricula.

## Methods

This study was approved by the Ethics Review committees of the IWK Health Centre, Capital Health, Dalhousie University, and St. Francis Xavier University.

### National Curriculum Review of Immunization-Related Content

A questionnaire was distributed to all Canadian medical, pharmacy, and four-year nursing baccalaureate programs regarding immunization-related content in their curriculum (Additional File 1). The questionnaire addressed three main content areas: (i) basic principles and practices of immunization; (ii) immunization clinical skills; and (iii) vaccine-preventable diseases. Programs were also asked to provide information on the total time allocated to immunization-related content, the scheduling of immunization content within the curriculum, and teaching methods used for content delivery. For each school, key contacts with responsibility for immunization content in the curriculum were identified through public directories, and were contacted directly. They were then sent an information letter and questionnaire and asked to participate in a 15 minute telephone interview.

### Assessment of Knowledge and Attitudes Regarding Immunization

A 77-item web-based self-administered questionnaire (VaxEd survey), developed using Remark™ Web Survey Software and validated at the Canadian Center for Vaccinology (Halifax, NS, Canada), was distributed to students in their final year of undergraduate training in medicine, nursing and pharmacy at two universities in Nova Scotia, Canada (the nursing schools have been identified as Nursing 1 and Nursing 2 to represent the different universities). Development and implementation of the survey followed the principles of Dillman [15]. Knowledge questions addressed general immunization information (schedules, routine guidelines), specific vaccines and vaccine preventable diseases, contraindications, and immunization recommendations in specific populations. Attitudinal statements, structured with a Likert-response scale from "strongly disagree" to "strongly agree" were used to evaluate opinions regarding various immunization themes (i.e. importance of immunization, multiple injections, vaccine myths and contraindications). Behaviour questions asked about the respondent's personal uptake of vaccines (i.e. influenza and tetanus). The purpose of surveying these populations was to establish baseline knowledge, attitudes and behaviors regarding immunization, reflecting the current curriculum content in the education programs.

### Statistical Analysis

Data were converted from Remark™ Survey Software files to Excel files. These were then imported to Stata 7.0, which was used for all statistical analysis. The national curriculum review was analyzed using descriptive statistics. For the web-based survey, descriptive statistics were used to estimate the proportion of respondents correctly answering the knowledge-based questions and those who had specific attitudes and behaviours regarding immunization and immunization education. Discrete variables were summarized using frequency counts, percentages, and 95% confidence intervals (CIs), and comparisons were made using Fisher's exact test and odds ratios (ORs). Continuous variables were summarized using means and 95% confidence intervals and comparisons were made using two-sided t-test and one-way ANOVA. Statistical significance was defined as a p-value ≤ 0.05.

## Results

### National Curriculum Review of Immunization-Related Content

Completed questionnaires regarding immunization-related content in health professional school curricula were received from 36% (32/89) of Canadian nursing schools. Additionally, 11 non-responding schools had identical shared curricula to one or more of the responding schools; therefore the data represented 48% (43/89) of nursing schools. Only 18% of medical schools returned completed questionnaires despite follow-up. 56% of pharmacy schools returned completed questionnaires.

**Figure 1:**
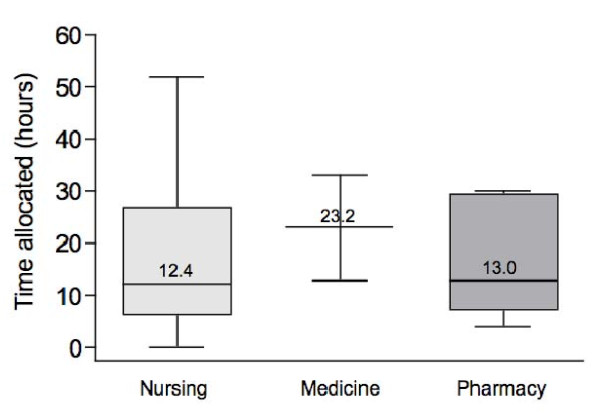
**Time allocated (hours) to immunization-related content in responding medical (n = 2), nursing (n = 30), and pharmacy (n = 5) programs**.* * Median values are shown for all programs. The upper and lower edges of the boxes represent the 75^th ^and 25^th ^percentiles, respectively.

A wide variation in the time allocated to immunization-related content was reported both between and within disciplines (Figure 1). Nursing programs reported the most variation with a minimum of less than one hour and a maximum of 52 hours. Nursing programs during which trainees participated as immunizers in public or occupational immunization campaigns had significantly more time allocated in the curriculum for immunization-related content (mean 17 h vs 3 h). There was also a wide-degree of variation in methods of teaching both between and within disciplines with no particular pattern.

**Figure 2:**
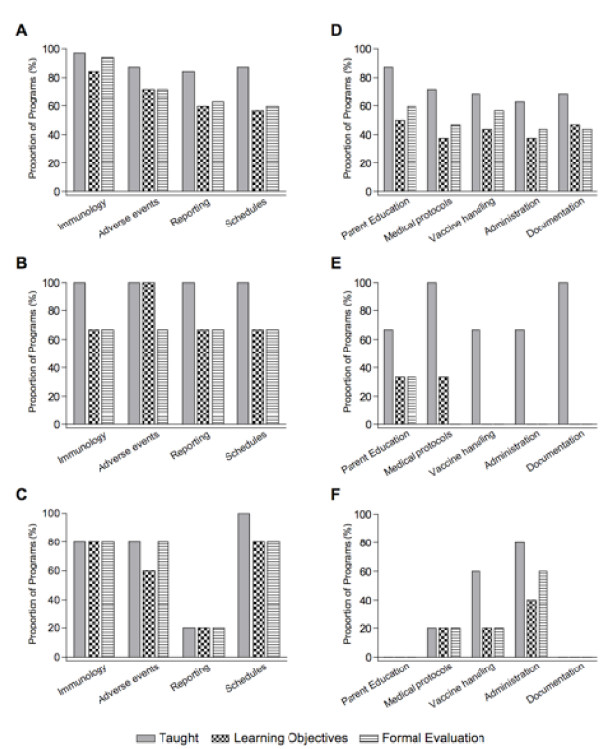
**Curriculum content related to immunization principles and practices: (A) Nursing; (B) Medicine; (C) Pharmacy, and curriculum content related to immunization clinical skills: (D) Nursing; (E) Medicine; (F) Pharmacy**.

Most programs reported the inclusion of some content regarding vaccine preventable diseases, immunization principles and practices, and clinical skills but there was considerable variability in the reported content both between and within disciplines (Figure 2A, B, C). All schools were also asked whether they teach, have specific learning objectives, and formally evaluate immunization-related clinical skills. Overall, programs reported less curriculum content related to clinical skills than to immunization principles and practices (Figure 2D, E, F).

Additionally, all schools were asked whether they teach, have specific learning objectives for, and formally evaluate content related to eleven different vaccine-preventable diseases. Nursing and pharmacy programs reported a lower proportion of curriculum content associated with specific learning objectives and formal evaluation. Medical programs reported specific learning objectives and formal evaluation associated with all of the curriculum content related to vaccine preventable diseases.

### Assessment of Knowledge and Attitudes Regarding Immunization

The 77-item VaxEd survey was sent to 353 health professional students in their final year of nursing, medicine, and pharmacy in Nova Scotia, Canada. The overall response rate was 147/353 (42%). Among programs surveyed, the response rate varied from 24% to 70.0%: 57/92 (70%) Nursing 1; 24/90 (26.7%) Nursing 2; 21/88 (23.9%) Medicine; and 45/83 (54.2%) Pharmacy. The majority of respondents were 20-30 years old (89%) and female (86%).

**Figure 3:**
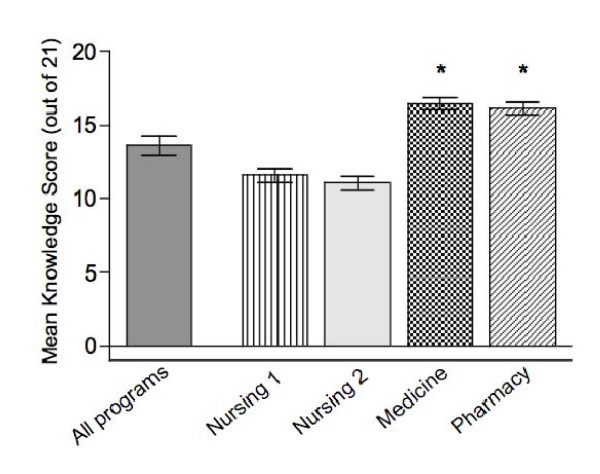
**Mean knowledge scores (out of 21) and comparison by program**.

There was significant variation between programs in immunization knowledge (Figure 3) with mean knowledge scores ranging from 11.1/21 to 16.4/21 (p < 0.001). Medicine and pharmacy respondents had significantly higher mean knowledge scores than nursing respondents (Table 1).

**Table 1:** Responses to selected knowledge questions including comparison of response by program.

	All programs (%)	Nursing 1 (%)	Nursing 2 (%)	Medicine (%)	Pharmacy (%)	Association with program (p-value)
Mild illness, with fever, is a reason to withhold vaccination						
*False	71 (49.0)	14 (25.5)	2 (8.3)	17 (81.0)	38 (84.4)	
True	65 (44.8)	34 (61.8)	21 (87.5)	4 (19.0)	6 (13.3)	< .001
Don't Know	9 (6.2)	7 (12.7)	1 (4.2)	0	1 (2.2)	
Giving multiple vaccines at the same time can overload the immune system						
*False	118 (80.3)	39 (68.4)	14 (58.3)	21 (100)	44 (97.8)	
True	18 (12.2)	11 (19.3)	7 (29.2)	0	0	< .001
Don't Know	11 (7.5)	7 (12.3)	3 (12.5)	0	1 (2.2)	
Pneumococcal vaccination is contraindicated for asplenic patients						
*False	58 (39.5)	12 (21.1)	7 (29.2)	16 (76.2)	23 (51.1)	
True	15 (10.2)	7 (12.3)	2 (8.3)	3 (14.3)	3 (6.7)	< .001
Don't Know	74 (50.3)	38 (66.6)	15 (62.5)	2 (9.5)	19 (42.2)	
Varicella vaccine can prevent chicken pox or reduce the severity of the disease if given within 3-5 days of exposure						
False	46 (31.3)	12 (21.1)	14 (58.3)	11 (52.4)	9 (20.0)	
*True	60 (40.8)	22 (38.6)	5 (20.8)	4 (19.1)	29 (64.4)	< .001
Don't Know	41 (27.9)	23 (40.3)	5 (20.8)	6 (28.5)	7 (15.6)	
Children who have had culture positive pertussis disease should not receive pertussis-containing vaccines						
*False	40 (27.4)	8 (14.0)	8 (34.8)	13 (61.9)	11 (24.4)	
True	33 (22.6)	18 (31.6)	7 (30.4)	2 (9.5)	6 (13.3)	< .001
Don't Know	73 (50.0)	31 (54.4)	8 (34.8)	6 (28.6)	28 (62.2)	
Routine childhood vaccines can be given to a child taking antibiotics for an ear infection						
False	37 (25.2)	15 (26.3)	12 (50.0)	6 (28.6)	4 (8.9)	
*True	82 (55.8)	23 (40.4)	7 (29.2)	14 (66.7)	38 (84.4)	< .001
Don't Know	28 (19.0)	19 (33.3)	5 (20.8)	1 (4.8)	3 (6.7)	
Prior egg ingestion is a prerequisite for immunization with measles, mumps and rubella						
*False	77 (52.4)	25 (43.9)	7 (29.2)	16 (76.2)	29 (64.4)	
True	43 (29.3)	17 (29.8)	14 (58.3)	3 (14.3)	9 (20.00)	.003
Don't Know	27 (18.3)	15 (26.3)	3 (12.5)	2 (9.5)	7 (15.6)	
Pertussis vaccine can cause sudden infant death syndrome						
*False	98 (67.1)	33 (58.9)	14 (58.3)	20 (95.3)	31 (68.9)	
True	7 (4.8)	3 (5.4)	1 (4.2)	0	3 (6.7)	.009
Don't Know	41 (28.1)	20 (35.7)	9 (37.5)	1 (4.7)	11 (24.4)	
Current scientific evidence supports associations between vaccines and chronic conditions such as autism and multiple sclerosis						
*False	114 (77.6)	37 (64.9)	13 (54.2)	21 (100)	43 (95.6)	
True	17 (11.6)	10 (17.5)	6 (25.00)	0	1 (2.2)	< .001
Don't Know	16 (10.8)	10 (17.5)	5 (20.8)	0	1 (2.2)	

Correct responses are indicated by an asterix (*).

Significant correlation was observed between increased knowledge and positive attitudes (Table 2). Overall, only 21% of respondents felt that they had received adequate teaching about vaccines during their training; these respondents had higher mean knowledge scores than those who did not feel that they had received adequate training (16/21 vs 12.3/21; p < 0.001); likewise, knowledge scores were higher among the 16% of respondents who reported feeling comfortable responding to parent/patient concerns about vaccine side effects than among those who were not (15.8/21 vs 12.3/21; p < 0.001).

**Table 2:** Attitudes regarding immunization that were significantly correlated with mean knowledge scores

	Response (%) to attitude questions	Mean knowledge score (out of 21)	p-value
It is not necessary to immunize breastfed infants at 2 months of age			
	Strongly disagree/disagree (58.6%)	14.8	< 0.001*
	Neutral/agree/strongly agree (41.4%)	11.9	
It is necessary to restart a series of vaccines if a dose is missed or delayed			
	Strongly disagree/disagree (55.9%)	15.0	< 0.001*
	Neutral/agree/strongly agree (44.1%)	11.7	
Vaccines may cause chronic diseases and learning disorders because they contain small amounts of mercury, aluminum, and formaldehyde			
	Strongly disagree/disagree (77.9%)	14.4	< 0.001*
	Neutral/agree/strongly agree (22.1%)	11.1	
It is no longer necessary to immunize against polio as it is now a rare disease in Canada			
	Strongly disagree/disagree (72.1%)	14.1	0.014*
	Neutral/agree/strongly agree (27.9%)	12.4	
Getting my annual influenza vaccine is important			
	Strongly disagree/disagree/neutral (11.6%)	11.2	0.004*
	Agree/strongly agree (88.4%)	14.0	
Getting tetanus/diphtheria toxoid (Td) vaccine (every 10 years) is important			
	Strongly disagree/disagree/neutral (11.8%)	11.8	0.037*
	Agree/strongly agree (88.4%)	13.8	
Children should be offered varicella vaccine at 12 months of age			
	Strongly disagree/disagree/neutral (34.2%)	11.7	< 0.001*
	Agree/strongly agree (65.8%)	14.7	
It is important to encourage all healthcare workers to be immunized annually with influenza vaccine			
	Not important/somewhat unimportant/neutral (8.3%)	10.8	0.006*
	Somewhat important/very important (91.7%)	13.9	
It is important to ensure that your adult patients have received all their required adult vaccines			
	Not important/somewhat unimportant/neutral (4.1%)	10.3	0.025*
	Somewhat important/very important (95.9%)	13.8	
Routine immunization should be delayed in individuals with moderate to severe illness, with or without fever			
	Strongly disagree/disagree (23.8%)	15.3	0.008†
	Neutral (17.7%)	13.4	
	Agree/strongly agree (58.5%)	13.0	
Parental stress can be reduced by spreading necessary vaccines over several visits			
	Strongly disagree/disagree (40.8%)	14.4	0.041†
	Neutral (21.8%)	12.4	
	Agree/strongly agree (37.4%)	13.4	
I am not comfortable recommending vaccines which are not government funded			
	Strongly disagree/disagree (40.4%)	14.3	0.012†
	Neutral (38.4%)	12.5	
	Agree/strongly agree (21.2%)	14.4	
I received adequate teaching about vaccines during my training			
	Strongly disagree/disagree (57.5%)	12.3	< 0.001†
	Neutral (21.2%)	14.8	
	Agree/strongly agree (21.2%)	16.0	
I am comfortable responding to questions parents/patients have about vaccine side effects			
	Strongly disagree/disagree (55.2%)	12.3	< 0.001†
	Neutral (18.6%)	14.6	
	Agree/strongly agree (26.2%)	15.8	

* Two-step t-test

† One-way ANOVA

85% of the students surveyed indicated that they received annual influenza immunization. Individuals who did not receive annual influenza immunization were less likely to agree that un-immunized health care workers can spread influenza to their patients (p < 0.001); more likely to agree that a healthy person does not need influenza immunization (p = 0.003); less likely to agree that if a health care worker does not receive influenza immunization, it is a failure of duty (p < 0.001); and much less likely to agree that receiving influenza immunization is important to them (p < 0.001). Also, this group had significantly lower mean knowledge scores than respondents who received annual influenza immunization (11.8/21 vs 13.2/21; p = .015).

## Discussion

A Canadian survey regarding preventative vaccines carried out in 2002 indicates that although support of vaccines among Canadians is broad, it is not very robust [12]. Concerns about vaccine safety in general (56%) and especially safety of new vaccines (43%) were quite broadly distributed. Another study indicated that attitudes, beliefs, and behaviours regarding vaccine safety concerns contribute substantially to under-immunization in the US [3]. It was shown that concerns were increasing amongst both parents of under-immunized and fully immunized children, as well as providers, suggesting the potential for further decreased coverage and an increase in disease outbreaks.

It is important therefore that providers are aware of immunization guidelines and vaccine safety issues, both real and perceived, and are able to communicate this information to patients and parents. Although knowledge and attitudes of health care providers have a significant impact on the frequency with which vaccines are offered and accepted, many health care providers are ill-equipped to make educated recommendations to their patients about the merits and risks of vaccines [7]. Possible reasons for this, as identified in the present survey, could be the lack of consistency in immunization education across Canada and significant gaps in knowledge coupled with worrisome attitudes regarding immunization among health professional trainees.

The national curriculum review demonstrated a wide degree of variation between health professional schools not only in the immunization-related content provided but also the amount of time allocated to immunization-related content, teaching methods used to deliver the content, and the degree to which students' knowledge of immunization content was evaluated. This variation existed between disciplines, as expected, but there was also considerable variation between programs of the same discipline. This lack of standardization results in variability in the quality of immunization education being received by health professional trainees. This is not a problem unique to Canada as similar results were reported in US-based studies [16,17].

Limitations of this study were identified. First, immunization-related content in most schools is scattered throughout the curriculum with little apparent communication or coordination, making the review challenging. Second, hardcopies of specific learning objectives and test questions were not obtained, potentially leading to an overestimation of what was taught. Finally, the low response rate, particularly from medical schools makes it likely that the selection bias limits the generalizeability of our conclusions and it is likely that our results may actually overestimate immunization curriculum content in health professional schools due to self- selection bias by schools with more extensive immunization-related curricula.

The VaxEd survey reflected the current curriculum delivered to graduating health professional trainees in nursing, medicine, and pharmacy. This revealed important insight into deficits in knowledge and worrisome attitudes and behaviours regarding immunization. These gaps in knowledge are concerning as they are not without precedent. In previous studies, physicians reported discomfort discussing misconceptions about adverse events following immunization with patients and many admitted to not being sure about the relationship between vaccines and chronic diseases [13,14]. This suggests that discomfort with immunization is an issue that is relevant at both a trainee and practicing level. Interestingly, 58% of students reported feeling that they had not received adequate training regarding immunization. This indicates that respondents had at least a degree of insight into the gaps in their own knowledge regarding immunization.

## Conclusion

In summary, despite the tremendous importance of physicians, nurses, and pharmacists in ensuring optimal delivery of immunization to Canadians, review of the curricula of undergraduate training programs revealed wide variability in immunization curriculum content and evaluation. This was reflected in the important knowledge gaps identified among trainees regarding vaccine indications/contraindications, adverse events, and safety and in the lack of satisfaction with immunization-related training reported by the majority of graduates.

Development and evaluation of a competency-based interprofessional immunization education program which could be adapted and integrated into formal educational curricula would contribute significantly to health professional training programs in Canada. The Immunization Competencies for Healthcare Professionals recently developed by the Professional Education Working Group of the Canadian Immunization Committee could provide a framework for use by educators to develop and evaluate immunization educational programs adapted to the needs of health professional trainees [18].

## Competing interests

This project is supported by an unrestricted educational grant from Sanofi Pasteur Canada Ltd. L Pelly's salary is supported by the Gladys Osman BSc. Medicine Studentship. S McNeil is supported by a Dalhousie University Faculty of Medicine Clinical Scholar's Award.

## Authors' contributions

All authors have contributed to the conception and design of the study, analysis of the data, manuscript development and final approval of the paper submitted.

## Pre-publication history

The pre-publication history for this paper can be accessed here:

http://www.biomedcentral.com/1472-6920/10/86/prepub

## Supplementary Material

Additional File 1 **Review of Vaccine-related content in Canadian Medical, Nursing, and Pharmacy Schools**. This file contains the survey tool used to assess the immunization-related content in Canadian Medical, Nursing, and Pharmacy schools. Click here for file
