# Supplementary material for: THE VAXED PROJECT: An Assessment of Immunization Education in Canadian Health Professional Programs
Source: BMC Med Educ. 2010 Nov 26;10:86. doi: 10.1186/1472-6920-10-86 (PMC3002370; doi:10.1186/1472-6920-10-86)
Supplement: Additional File 1 — Review of Vaccine-related content in Canadian Medical, Nursing, and Pharmacy Schools. This file contains the survey tool used to assess the immunization-related content in Canadian Medical, Nursing, and Pharmacy schools. [file 1472-6920-10-86-S1.PDF]

Review of Vaccine-related Content in Canadian Medical, Nursing, and Pharmacy Schools **25May2005**

1. What format is generally used for teaching? If multiple formats for teaching are used, please indicate the relative proportion of time spent using each format.

	Format (e.g. case-based learning, didactic lectures, simulated patients, etc.)
Pre-clinical training	
Clinical training	
Communication skills	

2. Immunization: basic principles and practices.

Subject	Is it taught? (Y/N)	By which department?	Is there a specific person? (If so, who?)	During what years of training?	Teaching format?	How much time is allotted?	Are there specific learning objectives?	Are students formally tested?
Immunology								
Adverse events following a vaccine								
Contraindications/precautions associated with immunization								
Immunization schedules (pediatric and adult)								

3. What resources are used to update the vaccine-related content? What resources are the students being encouraged to use? (e.g. books, websites, articles, etc.)

4. Clinical skills

Subject	Is it taught? (Y/N)	By which department?	Is there a specific person? (If so, who?)	During what years of training?	Teaching format?	How much time is allotted?	Are there specific learning objectives?	Are students formally tested?
Parent education (e.g. informed consent, screening, comforting restraints)								
Communication skills (risk/benefit communication)								
Medical protocols (e.g. anaphylaxis management)								
Vaccine handling (e.g. selecting correct equipment, proper vaccine handling and storage, cold chain)								
Administration (including multiple immunizations)								
Documentation and providing records (including adverse event reporting)								

5. Content on vaccine-preventable diseases (VPDs)

Subject	Is it taught? (Y/N)	By which department?	Is there a specific person? (If so, who?)	During what years of training?	Teaching format?	How much time is allotted?	Are there specific learning objectives?	Are students formally tested?
**Measles**								
Microbiology and pathophysiology								
Clinical features of the disease								
Epidemiology and prevention (including indications for vaccine)								
**Mumps**								
Microbiology and pathophysiology								
Clinical features of the disease								
Epidemiology and prevention (including indications for vaccine)								
**Rubella**								
Microbiology and pathophysiology								
Clinical features of the disease								
Epidemiology and prevention (including indications for vaccine)								
**Diphtheria**								
Microbiology and pathophysiology								
Clinical features of the disease								
Epidemiology and prevention (including indications for vaccine)								
**Tetanus**								
Microbiology and pathophysiology								
Clinical features of the disease								
Epidemiology and prevention (including indications for vaccine)								

**Poliomyelitis**								
Microbiology and pathophysiology								
Clinical features of the disease								
Epidemiology and prevention (including indications for vaccine)								
***Haemophilus influenzae* type b**								
Microbiology and pathophysiology								
Clinical features of the disease								
Epidemiology and prevention (including indications for vaccine)								
**Influenza**								
Microbiology and pathophysiology								
Clinical features of the disease								
Epidemiology and prevention (including indications for vaccine)								
**Pneumococcal disease**								
Microbiology and pathophysiology								
Clinical features of the disease								
Epidemiology and prevention (including indications for vaccine)								
**Hepatitis B**								
Microbiology and pathophysiology								
Clinical features of the disease								
Epidemiology and prevention (including indications for vaccine)								
**Pertussis**								
Microbiology and pathophysiology								
Clinical features of the disease								

Epidemiology and prevention (including indications for vaccine)								

Notes:
